# Aerobic and Facultative Bacterial Spectrum and Antimicrobial Susceptibility Patterns in Pediatric Acute Appendicitis: A Prospective Local Surveillance Study

**DOI:** 10.1155/ijm/4159509

**Published:** 2026-07-30

**Authors:** Yan Peng, Yulu Zhang, Lei Liu, Mengqi Li, Huaiwei Zhang

**Affiliations:** ^1^ Clinical Laboratory, The Affiliated Second Hospital of Fuyang Normal University, Fuyang Normal University, Fuyang, Anhui, China, fync.edu.cn; ^2^ Intensive Care Unit, The Affiliated Second Hospital of Fuyang Normal University, Fuyang Normal University, Fuyang, Anhui, China, fync.edu.cn

**Keywords:** aerobic bacteria, antimicrobial resistance, antimicrobial susceptibility, *E. coli*, extended-spectrum *β*-lactamase, facultative bacteria, pediatric acute appendicitis

## Abstract

**Background:**

Acute appendicitis is one of the most common surgical emergencies in children. In complicated appendicitis, timely empirical antimicrobial treatment is important, but antimicrobial selection is increasingly affected by regional variation in pathogen distribution and resistance. This study characterized the aerobic and facultative bacterial isolates recovered from intraoperative purulent specimens obtained from pediatric patients with acute appendicitis and described their antimicrobial susceptibility patterns.

**Methods:**

This prospective observational study enrolled 70 consecutive pediatric patients who underwent appendectomy for acute appendicitis at the Second Affiliated Hospital of Fuyang Normal University between January 2024 and January 2025. Intraoperative purulent material was collected using sterile swabs and processed by routine aerobic culture methods. No dedicated anaerobic culture or culture‐independent molecular analysis was performed. Bacterial identification was conducted using the VITEK 2 Compact system. Antimicrobial susceptibility testing was performed using broth microdilution and interpreted according to the applicable Clinical and Laboratory Standards Institute criteria. Extended‐spectrum *β*‐lactamase production among *Escherichia coli* isolates was confirmed using the double‐disk synergy test. Appendicitis was operationally categorized as uncomplicated or complicated according to operative and pathological findings.

**Results:**

Aerobic or facultative bacterial growth was detected in 62 of 70 specimens, corresponding to a culture‐positive rate of 88.6% (95% confidence interval, approximately 78.2%–94.0%). A total of 62 isolates were recovered from the 62 culture‐positive specimens, and no polymicrobial aerobic or facultative growth was recorded using the applied culture strategy. Gram‐negative organisms accounted for 61 of 62 isolates (98.4%). *E. coli* was the predominant isolate (46/62, 74.2%), followed by *Pseudomonas aeruginosa* (9/62, 14.5%). Nineteen of the 46 *E. coli* isolates (41.3%) were ESBL producers. *E. coli* retained high in vitro susceptibility to amikacin and imipenem but showed substantial resistance to ceftriaxone and trimethoprim–sulfamethoxazole. All nine *P. aeruginosa* isolates were susceptible to the antimicrobial agents tested. Based on the revised clinical classification, nine patients had uncomplicated appendicitis and 61 had complicated appendicitis.

**Conclusions:**

The organisms recovered with the present culture strategy were predominantly aerobic or facultative gram‐negative bacteria, especially *E. coli*, and ESBL production was frequent among *E. coli* isolates. These findings provide local surveillance information that may support antimicrobial stewardship and the reassessment of empirical coverage in pediatric appendicitis. However, because anaerobic culture, treatment comparisons, and comprehensive outcome analyses were not performed, the study does not characterize the complete appendiceal microbiota or establish the superiority of a specific empirical antimicrobial regimen.

## 1. Introduction

Acute appendicitis is among the most common causes of emergency abdominal surgery in children [1]. Pediatric patients may initially present with nonspecific symptoms and can progress to gangrene, perforation, localized peritonitis, or generalized peritonitis. Timely diagnosis, source control, and appropriate perioperative antimicrobial treatment are therefore important for reducing infectious complications and improving clinical outcomes [2, 3].

Appendectomy remains a standard treatment for most children with acute appendicitis, although nonoperative treatment may be considered in selected patients. Antimicrobial therapy is an essential component of perioperative care [4]. In uncomplicated appendicitis, perioperative prophylaxis or a short antimicrobial course is generally sufficient. In complicated appendicitis, including gangrenous or perforated disease and appendicitis associated with peritonitis, broader empirical coverage is commonly required while source control is achieved [5].

Appendicitis‐associated intra‐abdominal infection is characteristically polymicrobial. Both facultative gram‐negative organisms, such as *E. coli*, and anaerobic organisms, particularly species of the *Bacteroides fragilis* group, may contribute to infection [6]. The organisms recovered in a clinical study depend substantially on the specimen type, transport conditions, culture media, atmospheric conditions, and whether culture‐independent molecular techniques are used [7]. Consequently, studies based only on routine aerobic culture should not be interpreted as describing the complete appendiceal microbiota.

Increasing antimicrobial resistance among enteric gram‐negative bacteria represents an additional challenge in the management of intra‐abdominal infection [8, 9]. Of particular concern is the emergence of extended‐spectrum *β*‐lactamase (ESBL)–producing Enterobacterales. ESBL‐producing *E. coli* may be resistant to third‐generation cephalosporins and other commonly used *β*‐lactam agents, potentially reducing the reliability of some traditional empirical regimens [10]. Nevertheless, microbiological resistance data alone cannot determine the superiority of a specific treatment regimen because clinical effectiveness also depends on disease severity, source control, pharmacokinetic and pharmacodynamic factors, and patient outcomes.

Pediatric appendicitis‐specific surveillance data remain limited in many regional healthcare systems [11]. The present prospective study investigated the aerobic and facultative organisms recovered from intraoperative purulent specimens collected from pediatric patients with acute appendicitis. The primary objectives were to describe organism distribution and antimicrobial susceptibility patterns. Secondary exploratory objectives were to examine whether culture positivity, major pathogen distribution, and ESBL‐producing *E. coli* differed between uncomplicated and complicated appendicitis [12].

## 2. Materials and Methods

### 2.1. Study Design and Setting

This prospective observational study enrolled consecutive pediatric patients diagnosed with acute appendicitis between January 2024 and January 2025 at the Second Affiliated Hospital of Fuyang Normal University. All enrolled patients underwent appendectomy, during which intraoperative purulent material was collected for microbiological analysis.

The study was designed primarily as a local descriptive surveillance study. It did not compare antimicrobial treatment regimens and was not designed or powered to establish the clinical superiority of any empirical antibiotic.

### 2.2. Inclusion and Exclusion Criteria

Patients were eligible if they (1) met the institutional clinical, laboratory, imaging, operative, and pathological criteria for acute appendicitis; (2) underwent appendectomy during the study period; and (3) had an intraoperative purulent specimen available for microbiological examination.

Patients were excluded if they had (1) a history of previous abdominal surgery; (2) a concurrent clinically apparent infection at admission; or (3) an unavailable or inadequately labeled intraoperative specimen.

### 2.3. Ethical Approval

The study was approved by the Medical Ethics Special Committee of Fuyang Normal University (Approval Number: HSR‐26‐000251). Written informed consent was obtained from the legal guardians of all participants. All procedures were conducted in accordance with institutional requirements and the Declaration of Helsinki.

### 2.4. Specimen Collection and Transport

Purulent specimens were aseptically collected intraoperatively using sterile dry swabs during appendectomy and placed in sterile containers, transported to the clinical microbiology laboratory within 60 min. For simple appendicitis cases (*n* = 9), where free pus was not abundant, material was collected from the appendiceal serosal surface and/or the pericecal recess where minimal exudate was identified intraoperatively, ensuring specimen availability across all subtypes. Specimens were inoculated onto blood agar and MacConkey agar using the quadrant streak method and incubated at 35°C for 18–24 h. Chocolate agar was incubated at 35°C in 5% CO_2_.

The use of swabs rather than aspirated pus or peritoneal fluid reflected the routine intraoperative sampling practice at the participating institution during the study period. Swab sampling may reduce bacterial recovery, particularly for anaerobes, and may introduce preanalytical variability. This limitation was considered when interpreting culture‐negative specimens and organism distribution.

### 2.5. Scope of Microbiological Culture

Specimens were inoculated onto blood agar, MacConkey agar, and chocolate agar plates supplied by Hefei Tianda Diagnostic Reagents Co. Ltd. The applied protocol was intended to recover aerobic and facultative organisms.

No dedicated anaerobic transport system, anaerobic culture media, anaerobic incubation, or 16S rRNA gene sequencing was used. Therefore, the results represent the spectrum of recoverable aerobic and facultative organisms under the applied laboratory conditions and should not be interpreted as the complete microbiological spectrum of pediatric appendicitis.

### 2.6. Bacterial Identification

Bacterial isolates were identified using the VITEK 2 Compact automated system (bioMérieux, France) with GP and GN identification cards. When uncommon organisms were identified, the corresponding laboratory records and identification confidence were reviewed.

The unusual isolates, including *Comamonas testosteroni* and *Enterococcus avium*, were interpreted cautiously. Because confirmatory molecular identification and replicate cultures were not performed, their classification as true pathogens rather than transient colonizers or contaminants could not be established solely from a single swab culture.

### 2.7. Antimicrobial Susceptibility Testing

Minimum inhibitory concentrations were determined using broth microdilution. Interpretive criteria followed the CLSI standards applicable during the study period. Results were categorized as susceptible, intermediate, or resistant. For antimicrobial agents for which an intermediate category was not applicable, the corresponding table cell was recorded accordingly.

ESBL production in *E. coli* isolates was confirmed using the double‐disk synergy test. Susceptibility results were reported as numbers and percentages using the number of isolates tested against each antimicrobial agent as the denominator.

### 2.8. Classification of Appendicitis Severity

To improve clinical clarity and avoid overlapping pathological categories, patients were classified into two principal groups:

1. Uncomplicated appendicitis: inflamed or suppurative appendicitis without gangrene, perforation, periappendiceal abscess, phlegmon, or localized/generalized peritonitis.
2. Complicated appendicitis: appendicitis associated with gangrenous change, perforation, periappendiceal abscess or phlegmon, localized peritonitis, diffuse/generalized peritonitis, or extensive purulent contamination.

The original postoperative descriptions were retained in the source database but were mapped to the above categories for analysis. Based on the reported pathological findings, nine patients were classified as having uncomplicated appendicitis and 61 as having complicated appendicitis.

The original categories were mapped as follows: acute simple appendicitis: uncomplicated, unless an operative complication was documented; acute appendicitis with peritonitis: complicated; acute suppurative appendicitis with diffuse peritonitis: complicated; acute appendicitis with perforation: complicated; acute appendicitis with perforation and diffuse peritonitis: complicated; and acute gangrenous appendicitis with perforation: complicated. Because operative and pathological terminology may differ, discrepancies were resolved by prioritizing the operative evidence of perforation, gangrene, abscess, phlegmon, or peritonitis.

### 2.9. Statistical Analysis

Data were analyzed using SPSS Version 25.0 (IBM Corp., Armonk, New York, United States).

Continuous variables were assessed for distribution and are presented as mean ± standard deviation or median with interquartile range, as appropriate. Categorical variables are presented as numbers and percentages. Exact or Wilson 95% confidence intervals were calculated for key microbiological proportions.

The following exploratory comparisons were prespecified:

1. Culture‐positive versus culture‐negative patients;
2. Uncomplicated versus complicated appendicitis;
3. ESBL‐producing versus non‐ESBL–producing *E. coli* infections;
4. Distribution of major organisms according to appendicitis severity.

The chi‐square test or Fisher′s exact test was used for categorical variables, depending on expected cell counts. For contingency tables larger than 2 × 2 with sparse data, the Fisher–Freeman–Halton exact test was used where available. Continuous variables were compared using the independent‐samples *t* test or Mann–Whitney *U* test, as appropriate.

All tests were two‐sided. A *p* value below 0.05 was regarded as statistically significant. Since the subgroup analyses were exploratory and involved small numbers, effect estimates, denominators, and confidence intervals were emphasized, and the results were not used to make definitive therapeutic recommendations.

## 3. Results

### 3.1. Patient Demographics and Clinical Characteristics

A total of 70 pediatric patients were included. Their demographic, clinical, operative, and postoperative characteristics are summarized in Table 1. Nine patients (12.9%) were categorized as having uncomplicated appendicitis, whereas 61 (87.1%) met the operational criteria for complicated appendicitis.

**Table 1:** Baseline demographic and clinical characteristics (*n* = 70).

Characteristic	Value
Age, years, median (IQR)	8.0 (5.0–11.0)
Age group, *n* (%)	
≤ 5 years	16 (22.9)
6–12 years	41 (58.6)
≥ 13 years	13 (18.6)
Sex, male/female	43/27
Duration of symptoms before admission, hours, median (IQR)	36 (24–60)
Prior antibiotic exposure (before specimen collection), *n* (%)	14 (20.0)
Operative approach—laparoscopic/open	64/6
Postoperative infectious complications, *n* (%)	7 (10.0)
Surgical site infection	4 (5.7)
Intra‐abdominal abscess	2 (2.9)
Readmission for infection	1 (1.4)
Length of hospital stay, days, median (IQR)	6 (4–8)
Postoperative antibiotic regimen, *n* (%)	
Third‐generation cephalosporin–based	31 (44.3)
*β*‐lactam/*β*‐lactam inhibitor (piperacillin–tazobactam etc.)	22 (31.4)
Carbapenem‐based	9 (12.9)
Others	8 (11.4)

*Note:* Prior antibiotic exposure: mainly oral cephalosporins or amoxicillin–clavulanate prescribed at referring clinics. Postoperative complications were recorded during the same admission and within 30 days of surgery.

### 3.2. Distribution of Pathological Subtypes

The distribution of postoperative pathological subtypes is delineated in Table 2. Acute appendicitis with peritonitis constituted the predominant subtype (47/70, 67.1%). Collapsing these into the simplified uncomplicated/complicated framework revealed nine (12.9%) uncomplicated and 61 (87.1%) complicated cases.

**Table 2:** Pathological subtypes of pediatric acute appendicitis (*n* = 70).

Pathological subtype	Number of cases (*n*)	Percentage (%)
Acute appendicitis with peritonitis	47	67.1
Acute simple appendicitis	9	12.9
Acute suppurative appendicitis with diffuse peritonitis	5	7.1
Acute appendicitis with perforation	4	5.7
Acute appendicitis with perforation and diffuse peritonitis	4	5.7
Acute gangrenous appendicitis with perforation	1	1.4
Total	70	100.0

*Note:* For subgroup analyses, “uncomplicated” = acute simple appendicitis (*n* = 9); “complicated” = all remaining subtypes (*n* = 61), encompassing perforation, peritonitis, gangrenous change, or extensive purulent contamination.

### 3.3. Pathogen Distribution in Intraoperative Specimens

Pathogens were isolated from 62/70 specimens, yielding a culture‐positive rate of 88.6%. Among these, gram‐negative bacteria accounted for 61/62 isolates (98.3%), whereas only one gram‐positive isolate (1.6%) was identified. *E. coli* was the predominant pathogen (46/62, 74.2%), followed by *P. aeruginosa* (9/62, 14.5%) (Table 3). Polymicrobial growth (≥ 2 organisms) occurred in 5/70 (7.1%) specimens.

**Table 3:** Pathogen distribution among 62 recovered isolates.

Pathogen	Number of isolates (*n*)	Percentage (%)
*Escherichia coli*	46	74.2
*Pseudomonas aeruginosa*	9	14.5
*Proteus mirabilis*	2	3.2
*Comamonas testosteroni*	1	1.6
*Enterococcus avium*	1	1.6
Other isolate(s)^a^	3	4.8
Total	62	100.0

^a^Other: *Klebsiella oxytoca* (1), *Enterobacter cloacae* (1), and *Citrobacter freundii* (1).

### 3.4. Antimicrobial Susceptibility Profile of *E. coli*

Among 46 *E. coli* isolates, 19 (41.3%; 95% CI: 27.4%–56.3%) were confirmed ESBL producers. The susceptibility profile, *E. coli*, exhibited universal susceptibility to imipenem and ertapenem (0% resistance) and high susceptibility to amikacin (97.8% susceptible). Marked resistance was observed against cotrimoxazole (69.6% resistant) and ceftriaxone (47.8% resistant). Cefepime resistance was 32.6% (Table 4).

**Table 4:** Antimicrobial susceptibility profile of *E. coli* isolates (*n* = 46).

Antibiotic	Susceptible (S), *n* (%)	Intermediate (I), *n* (%)	Resistant (R), *n* (%)
Amikacin	45 (97.8)	1 (2.2)	0 (0)
Imipenem	46 (100)	0 (0)	0 (0)
Ertapenem	46 (100)	0 (0)	0 (0)
Cefoperazone	45 (97.8)	1 (2.2)	0 (0)
Tigecycline	45 (97.8)	0 (0)	1 (2.2)
Piperacillin	44 (95.7)	0 (0)	2 (4.3)
Cefoxitin	37 (80.4)	3 (6.5)	6 (13.0)
Ceftazidime	27 (58.7)	6 (13.0)	13 (28.3)
Cefepime	31 (67.4)	0 (0)	15 (32.6)
Amoxicillin	31 (67.4)	8 (17.4)	7 (15.2)
Cefuroxime sodium	21 (45.7)	3 (6.5)	22 (47.8)
Cefuroxime axetil	25 (54.3)	3 (6.5)	22 (47.8)
Ceftriaxone	24 (52.2)	0 (0)	22 (47.8)
Levofloxacin	9 (19.6)	18 (39.1)	19 (41.3)
Trimethoprim–sulfamethoxazole	14 (30.4)	0 (0)	32 (69.6)

*Note:* Interpretive criteria: CLSI 2024 [15]. ESBL confirmed in 19/46 (41.3%) isolates.

### 3.5. Antimicrobial Susceptibility Profile of *P. aeruginosa*

All nine *P. aeruginosa* isolates were susceptible to the tested agents (Table 5). Intermediate susceptibility was noted for ticarcillin (2/9, 22.2%) and colistin (1/9, 11.1%). No carbapenem‐resistant *P. aeruginosa* strains were detected.

**Table 5:** Antimicrobial susceptibility profile of *P. aeruginosa* isolates (*n* = 9).

Antimicrobial agent	S (*n*)	I (*n*)	R (*n*)	S (%)	I (%)	R (%)
Amikacin	9	0	0	100.0	0.0	0.0
Imipenem	9	0	0	100.0	0.0	0.0
Meropenem	9	0	0	100.0	0.0	0.0
Cefepime	9	0	0	100.0	0.0	0.0
Ceftazidime	9	0	0	100.0	0.0	0.0
Cefoperazone	9	0	0	100.0	0.0	0.0
Piperacillin	9	0	0	100.0	0.0	0.0
Tobramycin	9	0	0	100.0	0.0	0.0
Levofloxacin	9	0	0	100.0	0.0	0.0
Ciprofloxacin	9	0	0	100.0	0.0	0.0
Ticarcillin	7	2	0	77.8	22.2	0.0
Colistin	8	1	0	88.9	11.1	0.0

### 3.6. Subgroup Analyses

#### 3.6.1. Pathogen and ESBL Distribution by Disease Severity

Stratifying by uncomplicated (*n* = 9) versus complicated (*n* = 61) appendicitis revealed distinct trends (Table 6). *E. coli* was isolated in 6/9 (66.7%) uncomplicated and 40/61 (65.6%) complicated cases (*p* > 0.99). *P. aeruginosa* was exclusive to complicated cases (9/61, 14.8% vs. 0%, *p* = 0.59). Among the 46 *E. coli* isolates (six from uncomplicated, 40 from complicated), ESBL production was observed in 1/6 (16.7%) uncomplicated versus 18/40 (45.0%) complicated cases (*p* = 0.22, Fisher′s exact test). While the trend favored complicated disease, the small number of uncomplicated *E. coli* isolates limited statistical power.

**Table 6:** Comparison of *E. coli* isolation and ESBL prevalence by severity.

Variable	Uncomplicated (*n*=9)	Complicated (*n*=61)	*p*
*E. coli* isolated, n/N (%)	6/9 (66.7)	40/61 (65.6)	> 0.99
*E. coli* isolates with ESBL+, n/N (%)	1/6 (16.7)	18/40 (45.0)	0.22
*P. aeruginosa* isolated, n/N (%)	0/9 (0)	9/61 (14.8)	0.59

#### 3.6.2. Resistance Patterns Stratified by ESBL Phenotype

Resistance rates for key antimicrobial agents differed significantly between ESBL‐producing (*n* = 19) and non‐ESBL–producing (*n* = 27) *E. coli* isolates (Table 7). ESBL producers exhibited dramatically higher resistance to cephalosporins: ceftriaxone (89.5% vs. 14.8%, *p* < 0.001), cefuroxime (89.5% vs. 18.5%, *p* < 0.001), cefepime (57.9% vs. 14.8%, *p* = 0.004), and ceftazidime (52.6% vs. 11.1%, *p* = 0.004). No significant differences emerged for cotrimoxazole or levofloxacin. Universal susceptibility to amikacin, imipenem, and ertapenem persisted regardless of ESBL status.

**Table 7:** Antimicrobial resistance rates (%) in ESBL‐producing versus non‐ESBL *E. coli*.

Antibiotic	ESBL+ (*n*=19) *R*%	Non‐ESBL (*n*=27) *R*%	*p* (Fisher′s)
Ceftriaxone	89.5	14.8	< 0.001
Cefuroxime sodium	89.5	18.5	< 0.001
Cefepime	57.9	14.8	0.004
Ceftazidime	52.6	11.1	0.004
Cotrimoxazole	73.7	66.7	0.73
Levofloxacin	57.9	29.6	0.11
Amikacin	0	0	—
Imipenem/ertapenem	0	0	—

## 4. Discussion

This prospective, single‐center surveillance study characterized the aerobic and facultative bacterial isolates recovered from intraoperative purulent swab specimens obtained from 70 children undergoing appendectomy for acute appendicitis [13]. Aerobic or facultative growth was detected in 88.6% of specimens, nearly all recovered isolates were gram‐negative, and *E. coli* was the predominant organism. Of particular epidemiological relevance, 41.3% of the *E. coli* isolates produced ESBLs, indicating a substantial local burden of resistance among appendicitis‐associated Enterobacterales.

These observations must be interpreted within the microbiological boundaries imposed by the study design. Acute appendicitis—particularly perforated disease and appendicitis accompanied by peritonitis—is conventionally regarded as a polymicrobial intra‐abdominal infection involving both facultative and obligately anaerobic organisms [14]. Anaerobes, most notably members of the *B. fragilis* group, are recognized contributors to the pathogenesis of complicated intra‐abdominal infection. Because the present protocol did not incorporate dedicated anaerobic transport, anaerobic incubation, or culture‐independent microbiological methods, the recovered isolates represent the cultivable aerobic and facultative fraction of the microbial community rather than the full microbiological spectrum of pediatric appendicitis [15].

The predominance of *E. coli* is consistent with its established role in appendiceal infection and other community‐acquired intra‐abdominal infections. Its relative abundance in the present cohort may nevertheless have been accentuated by the culture strategy, as Enterobacterales are readily recovered under routine aerobic conditions, whereas many obligate anaerobes require stringent collection, transport, and incubation procedures [16]. Accordingly, the absence of *Bacteroides* spp. and other anaerobic taxa should be regarded as nondetection under the applied laboratory conditions rather than evidence of their biological absence.

The culture‐positive rate of 88.6% indicates that routine culture recovered at least one aerobic or facultative organism from most specimens. This comparatively high yield does not, however, obviate the inherent limitations of swab‐based sampling. Aspirated pus or peritoneal fluid is generally preferable for the microbiological investigation of intra‐abdominal infection because it provides a larger and potentially more representative inoculum and is more amenable to anaerobic transport. Swabs may contain limited material, preferentially recover dominant or readily cultivable organisms, and be particularly susceptible to variability in sampling technique and transport time [17]. These factors may also explain why no specimen yielded more than one reported organism despite the well‐established polymicrobial nature of complicated appendicitis. The apparent monomicrobial recovery pattern should therefore be understood as a feature of the sampling and culture methodology rather than as evidence that the underlying infections were biologically monomicrobial [18].

The eight culture‐negative specimens warrant similarly cautious interpretation. Negative growth may reflect a low bacterial burden, antimicrobial exposure before specimen collection, insufficient specimen volume, transport‐related loss of viability, or the presence of anaerobic or fastidious organisms not recoverable under the applied conditions [19]. Culture‐negative appendicitis should not therefore be equated with microbiological sterility. If reliable patient‐level data on presampling antimicrobial exposure are available, comparison of culture‐positive and culture‐negative patients may help clarify whether previous treatment influenced culture yield. Any such analysis would remain exploratory, however, given the small number of culture‐negative cases and the consequent imprecision of effect estimates [20].

The 41.3% prevalence of ESBL production among *E. coli* isolates represents an important local surveillance signal. ESBL production commonly compromises the activity of ceftriaxone and related extended‐spectrum cephalosporins and is frequently accompanied by coresistance to other antimicrobial classes. The observed ceftriaxone resistance therefore supports periodic reassessment of institutional antibiograms and empirical treatment protocols, particularly for critically ill children and those with established risk factors for resistant Enterobacterales. These microbiological findings do not, however, establish the clinical superiority of carbapenems, aminoglycosides, or any other individual regimen [21]. Antimicrobial treatment was neither randomized nor prospectively compared, and the study did not evaluate treatment failure attributable to discordance between initial therapy and in vitro susceptibility.

Preservation of carbapenem activity remains a central antimicrobial stewardship priority. Although imipenem demonstrated high in vitro activity against the recovered *E. coli* isolates, the present findings do not support routine empirical carbapenem treatment for all children with appendicitis. Empirical selection should integrate clinical severity, adequacy of source control, recent antimicrobial exposure, previous colonization or infection with resistant organisms, local cumulative susceptibility data, allergy history, renal function, and institutional guidance [22]. Once microbiological results become available, treatment should be reassessed and narrowed whenever clinically appropriate.

Amikacin likewise retained high in vitro activity, but this finding should not be interpreted as support for aminoglycoside monotherapy. Aminoglycosides carry clinically relevant risks of nephrotoxicity and ototoxicity, and their use as standalone treatment would not provide dependable coverage of the broader polymicrobial and anaerobic spectrum expected in complicated intra‐abdominal infection [23]. Moreover, the present study was not designed to assess the adequacy of anaerobic coverage or the clinical effectiveness of amikacin‐containing regimens.

All nine *P. aeruginosa* isolates were susceptible to the agents tested. Although this result suggests preserved in vitro activity within the study cohort, the small number of isolates precludes robust inference regarding the wider local population. The recovery of *P. aeruginosa* from a subset of specimens also does not, in itself, justify routine empirical antipseudomonal therapy for all children with appendicitis. The need for such coverage should be determined by local epidemiology, illness severity, prior healthcare exposure, recent antimicrobial use, and patient‐specific risk factors.

The recovery of *C. testosteroni* merits particular consideration. This environmental, nonfermenting gram‐negative organism is an uncommon human pathogen, although sporadic cases of intra‐abdominal and bloodstream infection have been described. Its detection in a single operative swab may represent genuine low‐frequency infection, transient colonization, or contamination during specimen collection or processing [24]. Evidence supporting pathogenicity would include pure or predominant growth, substantial organism burden, repeated isolation, concordant blood cultures, and a clinically compatible response to directed therapy. In the absence of these corroborating data, its clinical significance remains indeterminate.

*E. avium* is likewise an unusual clinical isolate. Enterococci are constituents of the gastrointestinal microbiota and may contribute to intra‐abdominal infection in selected contexts, particularly severe, postoperative, or healthcare–associated disease [25]. Their recovery does not, however, imply a universal need for empirical enterococcal coverage in community‐acquired appendicitis. Given that only one gram‐positive isolate was identified and no confirmatory molecular testing was performed, the pathogenic relevance of *E. avium* in this cohort cannot be established with confidence [26].

The original pathological categories were consolidated because perforation, gangrene, suppuration, and peritonitis are overlapping clinical and pathological features rather than mutually exclusive disease states. Reclassification into uncomplicated and complicated appendicitis provides a more clinically interpretable framework and is better aligned with contemporary therapeutic decision‐making [27]. Nevertheless, the pronounced imbalance between the groups—nine uncomplicated and 61 complicated cases—substantially restricts severity‐stratified inference. Estimates derived from the uncomplicated subgroup are inherently imprecise and particularly vulnerable to sparse‐data bias.

Comparison of ESBL‐producing *E. coli* between uncomplicated and complicated appendicitis is clinically relevant but should be treated as exploratory. A higher observed prevalence in complicated disease would require cautious interpretation because of the small denominators and the potential influence of prior antimicrobial exposure, symptom duration, referral patterns, healthcare contact, and other unmeasured confounders [28]. Conversely, the absence of a statistically significant difference would not demonstrate equivalence between the severity groups, as the study was not adequately powered for that purpose. Effect estimates and confidence intervals are therefore more informative than reliance on statistical significance alone [29].

Taken together, the data provide contemporary local surveillance of aerobic and facultative bacterial isolates but do not establish a severity‐stratified therapeutic algorithm. Development of a robust empirical treatment framework would require a larger and more balanced cohort, reliable recovery of anaerobic organisms, detailed patient‐level antimicrobial exposure data, resistance comparisons across clinically defined severity groups, and linkage of microbiological findings to treatment and outcome measures [30]. The implications of the present study are therefore appropriately confined to institutional surveillance, antibiogram‐informed protocol review, and antimicrobial stewardship rather than prescriptive endorsement of a specific regimen.

### 4.1. Clinical Implications

The predominance of *E. coli* confirms that this organism remains a central target of empirical antimicrobial coverage for pediatric appendicitis at the study institution. The high proportion of ESBL‐producing isolates further suggests that continued reliance on third‐generation cephalosporins should be periodically reassessed against updated local susceptibility data, particularly in children with severe or complicated disease and in those with recognized risk factors for resistant Enterobacterales [31].

These findings should not be interpreted as supporting unrestricted escalation to carbapenems or other broad‐spectrum agents. Rather, they underscore the need for a risk‐adapted approach that reconciles the probability of adequate initial coverage with the ecological consequences of unnecessary broad‐spectrum antimicrobial exposure [32]. Empirical therapy should remain aligned with local and international guidance and should be reviewed promptly after source control and microbiological results become available.

The absence of anaerobic isolates has no bearing on whether anaerobic coverage is clinically required because anaerobic organisms were not adequately investigated. Treatment decisions must therefore account for the expected polymicrobial composition of appendicitis‐associated intra‐abdominal infection, including organisms beyond those recovered using the present culture protocol.

### 4.2. Strengths

The prospective design and consecutive enrollment over a predefined study period reduced some selection and ascertainment biases associated with retrospective laboratory series. Standardized automated identification and susceptibility testing enhanced consistency in isolate characterization. The study also provides contemporary institution‐specific data on ESBL production and antimicrobial susceptibility among *E. coli* isolates recovered from children with acute appendicitis—information that may be valuable for local antibiogram interpretation, protocol review, and stewardship planning.

### 4.3. Limitations

Several methodological constraints define the scope and interpretation of this study. Dedicated anaerobic culture and culture‐independent molecular analyses were not performed; consequently, the findings are restricted to aerobic and facultative organisms recoverable under the applied laboratory conditions and do not represent the complete appendiceal microbiota.

Specimen collection relied on sterile swabs rather than aspirated pus or peritoneal fluid. Swab specimens typically provide a smaller inoculum, are more sensitive to variability in sampling technique and transport time, and are poorly suited to preserving obligate anaerobes [33]. This sampling approach may have influenced both culture yield and the relative distribution of recovered organisms.

The absence of multiple reported isolates from individual specimens is also likely to reflect the restricted sampling and culture methodology. It should not be construed as evidence that pediatric appendicitis is intrinsically monomicrobial.

The single‐center design and modest cohort size limit external validity. In particular, only nine patients were categorized as having uncomplicated appendicitis, resulting in imprecise estimates and limited statistical power for comparisons according to disease severity. Sparse subgroup data also increase the possibility of unstable effect estimates and Type II error.

The study was therefore unable to determine reliably whether resistant or ESBL‐producing organisms were associated with antimicrobial escalation, postoperative intra‐abdominal abscess, surgical‐site infection, treatment failure, readmission, or prolonged hospitalization [34]. Residual confounding by previous antimicrobial exposure and disease severity also cannot be excluded.

Susceptibility results were derived from in vitro testing and should not be considered direct surrogates for clinical effectiveness. Outcomes in intra‐abdominal infection depend on multiple factors beyond laboratory susceptibility, including the timing and adequacy of source control, antimicrobial exposure at the site of infection, tissue penetration, disease severity, inoculum, and host characteristics [35].

No prospective comparison of antimicrobial regimens was undertaken, and the study was not designed to demonstrate the superiority of any empirical treatment strategy. Its findings should therefore be regarded as local epidemiological evidence to inform surveillance and stewardship, not as an independent prescribing algorithm.

### 4.4. Future Research

Future investigations should adopt multicenter, adequately powered designs and preferentially collect aspirated peritoneal fluid or pus using validated anaerobic transport systems. Parallel aerobic and anaerobic culture would provide a more representative account of appendicitis‐associated microbiology. Culture‐independent approaches, including 16S rRNA gene sequencing or metagenomic methods, could further delineate fastidious, low‐abundance, uncultured, or nonviable organisms that escape conventional culture [36, 37].

Prospective linkage of isolate‐level microbiological data with prior antimicrobial exposure, operative severity, source‐control adequacy, initial and definitive treatment, and clinically meaningful outcomes is particularly important. Relevant outcomes should include postoperative intra‐abdominal abscess, surgical‐site infection, antimicrobial escalation, treatment failure, readmission, and length of hospitalization [38].

Larger cohorts would also permit multivariable assessment of whether ESBL‐producing Enterobacterales or other resistance phenotypes are independently associated with complicated appendicitis and adverse postoperative outcomes. Severity‐specific or resistance phenotype–guided empirical treatment strategies should not be recommended until their clinical effectiveness, safety, and ecological consequences have been evaluated in appropriately designed comparative studies [39].

## 5. Conclusions

Routine aerobic culture of intraoperative purulent swab specimens from this pediatric appendicitis cohort predominantly recovered gram‐negative facultative organisms, with *E. coli* representing the principal isolate. ESBL production was detected in 41.3% of *E. coli* isolates, indicating a substantial local burden of resistance and supporting continued institution‐specific surveillance of antimicrobial susceptibility.

These findings may inform local antibiogram review, empirical protocol reassessment, and antimicrobial stewardship. Their clinical interpretation must nevertheless remain circumscribed by the study design. Obligate anaerobes were not adequately investigated, antimicrobial regimens were not comparatively evaluated, and microbiological resistance was not linked comprehensively to treatment outcomes. The results therefore neither characterize the complete microbiology of pediatric appendicitis nor demonstrate the superiority of any specific empirical antimicrobial regimen. Empirical treatment should continue to integrate disease severity, expected polymicrobial coverage, patient‐level resistance risk, local susceptibility data, source‐control adequacy, and established stewardship principles.

## Author Contributions

**Yan Peng:** funding acquisition, writing – original draft preparation, writing – reviewing and editing, project administration, supervision. **Yulu Zhang:** methodology, investigation, visualization, writing – reviewing and editing. **Lei Liu:** conceptualization, investigation, visualization, writing – reviewing and editing. **Mengqi Li:** data curation, funding acquisition, writing – original draft preparation, writing – reviewing and editing. **Huaiwei Zhang:** data curation, funding acquisition, writing – original draft preparation, writing – reviewing and editing, project administration.

## Funding

This study was supported by Natural Science Research Project of Fuyang Normal University (2022KYQD0016); Fuyang Normal University Outstanding Talent Education and Training Program (2025ZYRCJH02); Fuyang Normal University Practice Education Special Project (2025SJYRZX11); Fuyang Normal University Medical Research Special Project (2024FYNUEY19, 2025FYNUZL04); and Anhui Provincial Quality Engineering Project (2025jcjs099).

## Ethics Statement

This study was conducted in accordance with the principles of the Declaration of Helsinki and was approved by the relevant institutional ethics committee of the Fuyang Normal University . Patient‐identifying information was removed before analysis. Because this study used routinely collected clinical and microbiological data and involved no additional intervention, the requirement for informed consent was waived by the ethics committee.

## Consent

The authors have nothing to report.

## Conflicts of Interest

The authors declare no conflicts of interest.

## Data Availability

The datasets generated and/or analyzed during the current study are available from the corresponding authors on reasonable request, subject to institutional and ethical restrictions.
